# Analysis for Bopivirus B in goats in the Sichuan province, China using a novel TaqMan real-time polymerase chain reaction assay

**DOI:** 10.1186/s13028-024-00777-3

**Published:** 2025-03-17

**Authors:** Kehamo Abi, Youwen Yang, Chen Yang, Kegu Ji’e, Falong Yang

**Affiliations:** Key Laboratory of Veterinary Medicine of Sichuan Province, Southwest Minzu University, Chengdu, 610041 China

**Keywords:** Bopivirus B, Diarrhea, Goat, TaqMan-based real-time PCR

## Abstract

**Background:**

Bopivirus B is an emerging picornavirus that affects goats in China. This study aimed to establish a TaqMan real-time PCR assay for detecting Bopivirus B and conduct a preliminary survey of infection in six goat farms in Sichuan province, China. Specific primers and a probe targeting the 3D gene of Bopivirus B were designed, and the TaqMan-based real-time PCR assay was successfully established following the optimization of reaction conditions and components. A total of 257 goat fecal samples were collected from six farms in Sichuan and tested using the newly developed method.

**Results:**

The assay demonstrated a linear relationship between 2.73 × 10^3^ and 2.73 × 10^9^ copies/µL, with a high correlation coefficient (R^2^ = 0.999) and amplification efficiency of 109%. Additionally, the assay exhibited excellent specificity and reproducibility, with a detection limit of 27.3 copies/µL. The field positive rate of Bopivirus B was 100%, and a higher positive rate was observed in diarrheal fecal samples (33.72%) compared to non-diarrheal fecal samples (12.28%, *P* < 0.005), suggesting a potential association between Bopivirus B and goat diarrhea, with a widespread prevalence in goats in the Sichuan province. Furthermore, ten complete 3D genes sequences of Bopivirus B were obtained, and phylogenetic analysis showed that all Bopivirus B strains in this study were most closely related to two known Chinese Bopivirus strains based on nucleotide sequences of the 3D gene.

**Conclusions:**

This study developed a highly specific, repeatable, and sensitive TaqMan-based real-time PCR assay targeting the 3D gene for Bopivirus B detection, offering a valuable tool for the detection and epidemiological investigation of Bopivirus B. The prevalence of Bopivirus B was widespread in goats in China, with a close association observed between Bopivirus B and goat diarrhea.

## Background

Bopivirus is a member of the genus within the family *Picornaviridae* and currently contains one officially recognized species, as well as two candidate species, namely Bopivirus A, “Bopivirus B” and “Bopivirus C” (http://www.picornaviridae.com/). Bopivirus A has been shown to infect cattle [1], while “Bopivirus B” has been identified in goats and sheep [2], and “Bopivirus C” has been found in deer [3]. However, due to the absence of a cell culture system for Bopivirus isolation and limited epidemiological data, the pathogenic potential of this virus in goats remains unclear.

In 2021, Bopivirus B was first identified in diarrheal fecal samples from goats and sheep in Hungary [2]. Subsequently, Bopivirus B was also detected in goats from Sichuan province, China [4]. Currently, Bopivirus B has been classified into two genotypes, Bopivirus B1 and Bopivirus B2, based on the Bopivirus species classification criteria of the International Committee on Taxonomy of Viruses (ICTV) (https://talk.ictvonline.org). Four Bopivirus B genome sequences from goats and sheep are available in GenBank. The genome of Bopivirus B consists of a 5’ untranslated region (UTR), a large open reading frame (ORF), and a 3’ UTR region. It encodes viral structural proteins P1(VP4, VP2, VP3, and VP1), as well as non-structural proteins P2 (2 A, 2B, and 2 C) and P3 (3 A, 3B, 3 C, and 3D) [2]. Although the precise biological function of Bopivirus B proteins remains unclear, proteins such as VP4, VP2, and VP1 in several members of *Picornaviridaea* have been implicated in cellular receptor recognition, antigenic diversity, and viral pathogenesis [5–7].

Traditional RT-PCR methods are time-consuming, labor-intensive, and do not allow for high-throughput detection and quantitative analysis of the virus, hereby limiting relevant research such as epidemiological investigations. To address these limitations, our study aimed to develop a TaqMan-based real-time quantitative PCR (qPCR) assay targeting conserved segments of the 3D gene for precise diagnosis and quantification of Bopivirus B viral loads. Additionally, we evaluated the performance of the newly established method and investigated the prevalence of Bopivirus B in Southwest China by testing fecal samples from goats collected during the period of 2021–2023. Furthermore, we analyzed the genetic evolution of the Bopivirus B 3D gene to enhance our understanding of the epidemiology of Bopivirus B in China.

## Methods

### Viruses, bacteria, and clinical samples

A total of 11 virus species and five bacterial species known to potentially infect goats were selected for evaluating the specificity of the TaqMan-based real-time qPCR assay. The virus species included Bovine coronavirus (BCoV), Ovine rotavirus (ORV), Bovine viral diarrhea virus (BVDV), infectious bovine rhinotracheitis virus (IBRV), Caprine astrovirus (CapAstV), Bovine astrovirus (BoAstV), Ovine hungarovirus (OhuV), Caprine enterovirus (CEoV), Caprine kobuvirus (CKoV), Ovine kobuvirus (OKoV), and Orf virus (ORFV). The bacterial species consisted of *Pasteurella multocida* (*P. multocida*), *Salmonella enterica* CVCC528(*S. enterica*), *Mannheimia haemolytica(M. haemolytica)*, *Escherichia coli* K99 (*E. coli K99*) and *Proteus mirabilis* CVCC3847 (*P. mirabilis*). Nucleic acid samples of these viruses and bacteria were obtained and stored in our laboratory for use in this study.

**Table 1:** Reproducibility of the Bopivirus B real-time PCR

Concentration of standard plasmid (copies/µL)	Intra-assay variability		Inter-assay variability	
	Ct(X ± SD)	CV (%)	Ct(X ± SD)	CV (%)
2.73 × 10^5^	23.85 ± 0.26	1.09	24.00 ± 0.15	0.63
2.73 × 10^6^	20.56 ± 0.19	0.90	20.46 ± 0.39	1.89
2.73 × 10^7^	18.43 ± 0.32	1.75	18.53 ± 0.45	2.44

Ct: threshold cycle; X: average Ct value; SD: standard deviation; CV: coefficient of variation

A total of 86 rectal samples were collected using gloved hands from goats with diarrhea on a farm in Zigong County from June 2021 to December 2021. These samples were used for comparing detection methods. These samples were used for comparing detection methods.

Additionally, a total of 257 samples, including 86 diarrheal samples and 171 non-diarrheal samples, were collected from goats in six different farms in Sichuan between June 2021 and January 2022. The ages of the tested goats ranged from 2 days to 3 months old. Detailed information regarding the samples used for the case-control test is presented in Table 2.

**Table 2:** Detection of Bopivirus B from clinical samples

Regions	Farm number	Samples	Bopivirus detection rate		Total detection rate
			diarrheal samples	non-diarrheal samples	
Zigong	1	89	16.00%(4/25)	6.25%(4/64)	8.99%
Jintang	1	33	28.60%(6/21)	8.33%(1/12)	21.21%
Shuangliu	1	20	33.33%(5/15)	20.00%(1/5)	30.00%
Xinjin	2	96	64.71%(11/17)	16.46%(13/79)	25%
Lezhi	1	19	37.50%(3/8)	18.18%(2/11)	26.32%
Total	6	257	33.72%(29/86)	12.28%(21/171)	19.46%

Note: fecal samples were collected from goats in different regions; the detection of Bopivirus B from clinical samples was performed using a TaqMan real-time PCR assay established in this study

### RNA extraction and cDNA synthesis

Fecal samples were suspended in phosphate-buffered saline (PBS) at a 1:5 ratio and centrifuged at 5,000 × g for 8 min. The supernatant was filtered through a 0.22-µm filter to remove debris. Viral RNA was extracted from the filtered suspension and reverse transcribed using a commercially available kit (TaKaRa Bio Inc., Shiga, Japan), following the manufacturer’s instructions. Furthermore, viral DNA was also extracted from the suspension using the Viral DNA Kit (Omega Bio, USA) according to the manufacturer’s instructions. The isolated DNA was then stored at − 20 °C.

### TaqMan-based real-time qPCR assay

#### Primer design

The primers and probe were designed using Beacon Designer 8 software based on the complete 3D gene sequence of the four different Bopivirus strains available in GenBank. A 139 bp segment of the 3D gene sequence (position 6,814–6,952 nt in the Bopivirus genome sequence, GenBank accession: ON044229) was selected as the target for the TaqMan-based real-time qPCR assay. The primer sequences were as follows: F: 5’- GGTGAACAACCTCWTCAA − 3’, R: 5’- GTCCAAGTCTARCGGTATC − 3’, and the TaqMan probe sequence: FAM-5’- ATCACCATAGCARACCACAGCA − 3’- TAMRA.

#### Optimization of TaqMan-based real-time qPCR

To optimize the TaqMan-based real-time qPCR assay, different concentrations of components were tested and screened in a total volume of 25 µL. This included the forward and reverse primers at concentrations ranging from 0.5 µM to 4 µM, the probe at concentrations of 10 µM, and Premix Ex Taq DNA polymerase (5 U/µL) (TaKaRa Biotechnology, Dalian, China) at a volume of 12.5 µL.

#### Specificity of TaqMan-based real-time qPCR

To determine the specificity of the TaqMan-based real-time qPCR assay, 16 major pathogens associated with goat diarrhea were tested as targets. These include BCoV, ORV, BVDV, IBRV, CapAstV, BoAstV, OhuV, CEoV, CKoV, OKoV, ORFV, *P. multocida*, *S. enterica*, *M. haemolytica*, *E. coli K99*, and *P. mirabilis*.

#### Sensitivity of TaqMan-based real-time qPCR

A Bopivirus B standard plasmid was prepared, and its copy number was quantified as described previously [8]. Ten-fold serial dilutions of the standard plasmid were prepared in RNase-free water and used to evaluate the sensitivity of the TaqMan-based real-time qPCR assay. All samples were amplified under the optimized conditions.

#### Reproducibility of TaqMan-based real-time qPCR

To assess the reproducibility of the TaqMan-based real-time qPCR assay, six different dilutions of the standard plasmid (10^− 4^-10^− 9^) were used. The reproducibility of the assay was evaluated by analyzing the amplification results.

#### Comparison with RT-PCR

A total of 86 clinical diarrheal samples collected from goats on a farm in Zigong were simultaneously analyzed using the TaqMan-based real-time qPCR assay established in this study and the only available conventional RT-PCR method for detecting Bopivirus B [2]. The agreement between the two methods was calculated to assess their concordance.

### Screening for Bopivirus by TaqMan-based real-time qPCR

The TaqMan-based real-time qPCR assays established in this study were used to screen a total of 257 clinical samples (collected as described in Table 2) for the presence of Bopivirus.

### Complete 3D gene sequence amplification and analysis

Three pairs of primers were designed based on the four complete Bopivirus B 3D gene sequences and 25 Bopivirus B 3D gene fragments available in GenBank. The primers used for amplifying the Bopivirus B complete 3D genes from diarrheal fecal samples, which tested positive for Bopivirus B, are as follows: Bopivirus B F1: 5′-AATGGTACCATCGGTTTCGCGTCGC-3′;Bopivirus B R1: 5′-GCCGGCTCGGACTTTGGCCACTGGG-3′ (5741–6427 nt, GenBank accession: ON044229); Bopivirus B F2: 5′-GGCTCCGGAGACAGGACCTCATCAA-3′, Bopivirus B R2: 5′-CATCACCATAGCARACCACAGCAAT-3′ (6261–6914 nt, GenBank accession: ON044229); Bopivirus B F3: 5′-TGGTGAACAACCTCWTCAACAACGT-3′, Bopivirus B R3: 5′- TTCAGGCCAAATCAGTCAAATCGTG-3′ (6813–7361 nt, GenBank accession: ON044229).

The PCR products were cloned into the pMD19-T simple vector (TaKaRa Bio Inc.) for sequencing, and the resulting sequences were assembled using SeqMan software (version 7.0; DNASTAR, Madison, WI, USA). Sequence alignments and clustering were performed by using ClustalW in MEGA 7.0 software. Nucleotide and deduced amino acid sequences were analyzed using the MegAlign software (DNASTAR, Madison, WI, USA). A phylogenetic tree was constructed using the neighbor-joining method with bootstrap values calculated for 1,000 replicates.

## Results

### TaqMan-based real-time qPCR

#### Standard curve of TaqMan qPCR

The optimal amplification conditions for TaqMan qPCR were established using a 25 µL reaction volume consisting of 12.5 µL of Premix Ex Taq DNA polymerase (5 U/µL), 0.5 µL of each primer (10 µM), 1.5 µL of probe (10 µM), 1.5 µL of cDNA, and an appropriate volume of double-distilled water. The PCR amplification was performed for 40 cycles with the following temperature profile: 95 °C for 30 s, 95 °C for 5 s, 56 °C for 30 s.

**Fig. 1:**
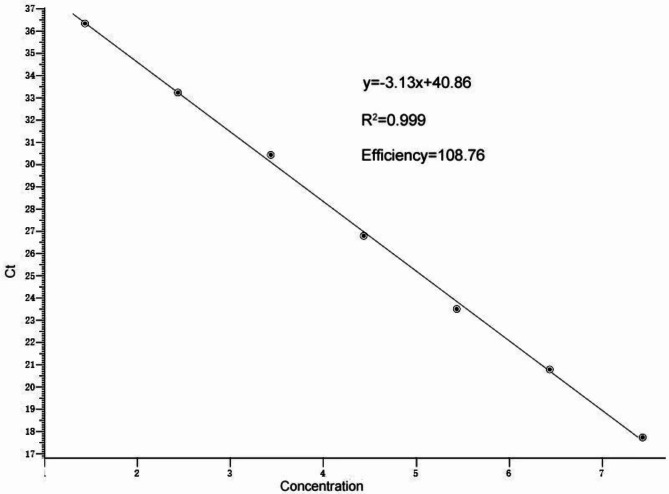
Standard curve of real-time PCR for Bopivirus B

The generated standard curve (as shown in Fig. 1) demonstrated a wide dynamic range of copy numbers from 2.73 × 10^3^~2.73 × 10^9^ copies/µL, with a linear correlation (R2) of 0.999 and efficiency of 109% between the Ct value and the logarithm of the plasmid copy number. The linear regressive equation was y = -3.13x + 40.86.

#### Specificity, sensitivity and reproducibility of TaqMan qPCR

**Fig. 2:**
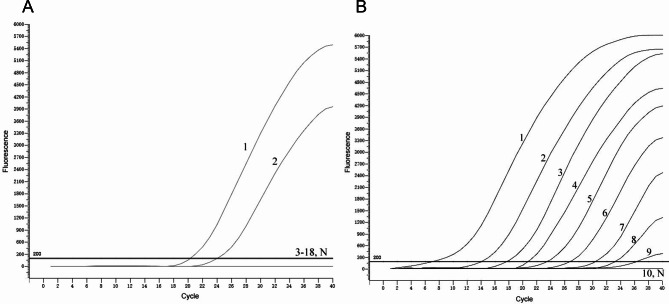
Specificity and sensitivity of TaqMan-based real-time PCR assay against Bopivirus B. **(A)** Specificity test for the real-time PCR. 1 ~ 2: Bopivirus B; 3 ~ 18: BCoV, ORV, BVDV, IBRV, CapAstV, BoAstV, OhuV, CEoV, CKoV, OKoV, ORFV, *P. multocida*, *S. enterica*, *M. haemolytica*, *E. coli K99*, and *P. mirabilis*; N: Negative control; **(B)** Sensitivity test for the real-time PCR. 1 ~ 10: 2.73 × 10^9^~2.73 × 10^0^; N: Negative control

The specificity testing results demonstrated that the TaqMan qPCR assay specifically amplified the target Bopivirus B sequence. Positive fluorescence amplification signals were only observed in samples containing Bopivirus B, while no amplification signal was detected in samples containing non-Bopivirus B goat-derived enteric viruses or nuclease-free water (Fig. 2A). The specificity analysis was independently repeated three times, and consistent results were obtained, confirming the high specificity of the developed TaqMan qPCR assay for Bopivirus B detection.

The sensitivity testing revealed that the assay could detect Bopivirus B at a limit of 27.3 copies/µL, indicating a high sensitivity for detecting the target virus (Fig. 2B). Three independent replications of the sensitivity test yielded identical results, demonstrating the consistent and reliable performance of the TaqMan qPCR assay.

The intra-assay and inter-assay coefficients of variation (CVs) were calculated to assess the repeatability and stability of the TaqMan qPCR assay. The results, presented in Table 1, showed that the intra-assay CVs ranged from 0.90 to 1.75%, indicating a high level of repeatability within the same test. Similarly, the inter-assay CVs ranged from 0.63 to 2.44%, indicating a high level of stability across different tests. These findings also confirm the consistent and reliable performance of the TaqMan qPCR assay.

### Comparison with RT-PCR

A total of 86 clinical diarrhea samples collected from goats were simultaneously tested for Bopivirus B using both the TaqMan real-time PCR method established in this study and the only available RT-PCR method. The results revealed that the detection rate of the TaqMan real-time PCR was 36.05%, which was significantly higher than the detection rate of RT-PCR method (12.79%, *P* < 0.05). Importantly, all the positive samples detected by the RT-PCR method were also detected by the TaqMan real-time PCR method. The Ct values for all positive samples ranged between 23 and 31, while the negative samples had Ct values greater than 40.

### Detection of Bopivirus in goats

Out of the 257 fecal samples, 50 (19.46%) were identified as Bopivirus B positive by TaqMan real-time qPCR established in this study. The average positive rate of Bopivirus B in diarrhea samples was 33.72% (29/86), whereas in health samples, the positive rate of Bopivirus B was12.28% (21/171) (*P* < 0.001). Furthermore, the positive rate of Bopivirus B in diarrhea samples from each farm was higher than that in healthy samples, as indicated in Table 2. These findings suggest a potential association between Bopivirus B and goat diarrhea.

### Molecular characterization of the 3D gene

The complete 3D genes were successfully cloned from 10 positive samples obtained from 5 farms in Sichuan Province (GenBank accessions OP272469- OP272478). These 10 3D genes were 1494 bp in length and encoded 497 amino acid residues. They exhibited a high degree of sequence similarity, sharing 95.2% to 99.5% nucleotide identity and 97.8% to 100% amino acid identity with each other. Furthermore, they showed 94.3% to 99% nucleotide identity and 96.6% to 99% amino acid identity with the four complete Bopivirus B 3D gene sequences avaiable in GenBank. Additionally, they displayed 71.2–97.7% nucleotide identity and 75.1–98.9% amino acid identity with the 25 Bopivirus B 3D gene fragments present in GenBank.

**Fig. 3:**
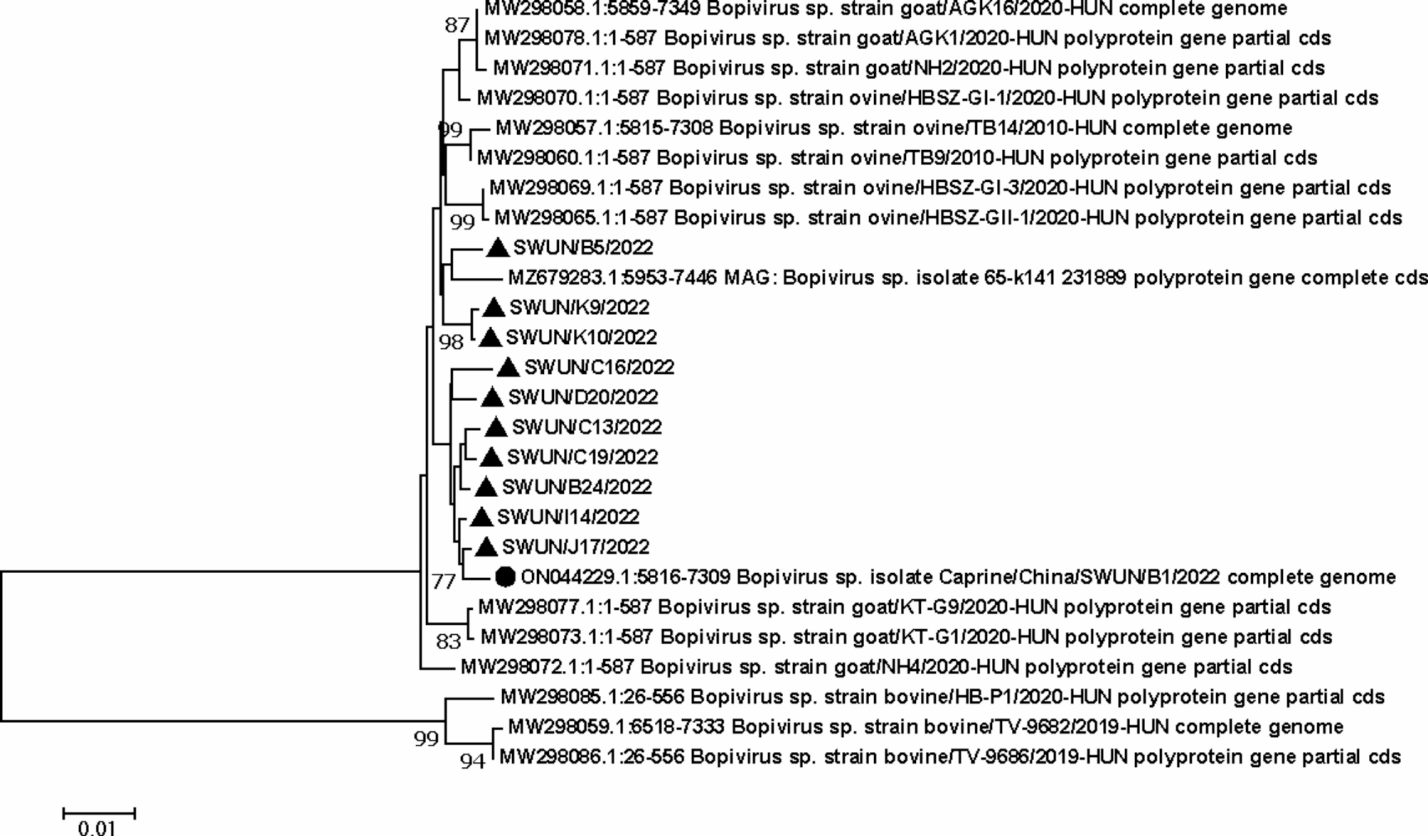
Neighbor-joining consensus tree for Bopivirus B 3D gene. Note: ▲marks the strain in this study, ●mark the Chinese strain previously cloned in our laboratory

A phylogenetic tree (Fig. 3) was constructed based on the Bopivirus B 3D gene sequences obtained in this study, along with 3D genes of other species of Bopivirus available in GenBank. The analysis revealed that seven Bopivirus B strains were most closely related to the 3D genes of the goat-derived Bopivirus B strain SWUN/B1/2022 (GenBank registration number ON044229) previously submitted by our laboratory to the GenBank database. The remaining 3D genes formed a distinct cluster with a Chinese sheep-derived Bopivirus strain 65-k141 (GenBank accession number MZ679283).

Notably, compared to the four complete Bopivirus B 3D gene sequences, the 10 Bopivirus B strains exhibited 21 amino acid unique substitutions in 3D gene.

## Discussion

### Development of TaqMan-based real-time qPCR assay

Most members of picornavirus are important pathogens that cause diarrhea in humans and various animals [9–11]. The 3D gene is commonly used as a target gene for virus detection because of its relatively conserved nature [2, 4, 12]. Bopivirus B, a newly discovered member of the picornavirus family, has emerged as a virus affecting goats in China [4]. Currently, only one specific RT-PCR assay has been reported for the detection Bopivirus B, targeting the 3D gene of a sheep-derived strain [2]. In this study, we developed a TaqMan-based real-time qPCR assay to detect Bopivirus B. The assay demonstrated high specificity and reproducibility, with a detection limit of viral RNA of 27.3 copies/µL. This assay significantly improves the detection rate of Bopivirus B in clinical samples compared to the previously reported RT-PCR assay. Therefore, the developed TaqMan-based real-time qPCR assay provides an effective tool for the detection, epidemiological investigation, and quantitative analysis of Bopivirus B.

### Detection of Bopivirus B in goats

Bopivirus B, a newly discovered picornavirus, has been detected in goats and sheep. However, limited information is available regarding its biological significance and prevalence. In this study, using the TaqMan-based real-time qPCR assay, we found that 19.46% of goat stool samples tested positive for Bopivirus B. The virus was detected in six farms in the Sichuan provinces of China. The positive rate of Bopivirus B in diarrhea samples was significantly higher (33.72%) than in non-diarrheal samples (12.28%, *P* < 0.005). Furthermore, the detection rate of Bopivirus B in the field was 100%, indicating a potential association between Bopivirus B and goat diarrhea, as well as its wide prevalence in goats in Sichuan Province. However, the pathogenic potential of Bopivirus B in animals remains unknown. It is worth noting that other members of the Picornaviridae family, such as enteroviruses and kobuviruses, are recognized as enteric viral pathogens [11, 13, 14]. Therefore, further investigations are necessary to expand the sample collection and explore the prevalence of Bopivirus B in China, as well as its pathogenicity in ruminants.

### Molecular characterization of the 3D gene

The 3D gene of picornavirus is relatively conserved, but it also exhibits genetic diversity based on geographical and host characteristics [15]. This makes the 3D gene a suitable target for molecular epidemiological analysis of viruses. Currently, only four complete Bopivirus B 3D gene sequences, including one Chinese strain, are avilable in GenBank. In this study, we obtained 10 complete 3D genes of Bopivirus B from positive samples collected from six farms in Sichuan Province. These sequences provide essential data for the development of detection methods and genetic evolution analysis of Bopivirus B. Phylogenetic analysis revealed that seven Bopivirus B strains in this study were closely related to the 3D genes of the goat-derived Bopivirus B strain SWUN/B1/2022, which was previously submitted by our laboratory to the GenBank database. The remaining 3D genes clustered with a Chinese sheep-derived Bopivirus strain 65-k141 on a separate branch of the tree, indicating a close relationship between goat-derived Bopivirus B strain and sheep-derived Bopivirus strain in China. Additionally, compared to the four complete Bopivirus B 3D gene sequences, the 10 Bopivirus B strains in this study exhibited 21 unique amino acid substitutions in 3D gene. Further investigations are needed to determine whether these unique amino acid mutations affect the function of the 3D gene in Chinese strains.

## Conclusions

This study successfully developed a TaqMan-based real-time fluorescent PCR assay for the detection of Bopivirus B using the 3D gene as the target. The assay demonstrated excellent specificity, repeatability, and sensitivity, offering a valuable tool for the detection and epidemiological investigation of Bopivirus B. The findings of this study revealed the wide prevalence of Bopivirus B in goats in China and highlighted the genetic diversity observed in the 3D gene of the virus. Further studies are warranted to elucidate the pathogenic potential and clinical significance of Bopivirus B in goats, as well as its potential impact on the livestock industry. Continued surveillance efforts, along with comprehensive molecular characterization and epidemiological investigations, will contribute to a better understanding of Bopivirus B and its implications for animal health.

## Data Availability

The datasets used and/or analysed during the current study are available from the corresponding author on reasonable request.

## Declarations

### Prior publication

Data have not been published previously.
